# Graph-Based Cooperative Localization Using Symmetric Measurement Equations

**DOI:** 10.3390/s17061422

**Published:** 2017-06-17

**Authors:** Dhiraj Gulati, Feihu Zhang, Daniel Clarke, Alois Knoll

**Affiliations:** 1 Robotics and Embedded Systems, Technische Universität München, Boltzmannstraße 3, 85748 Garching bei München, Germany; dhiraj.gulati@tum.de (D.G.); knoll@in.tum.de (A.K.); 2 School of Marine Science and Technology, Northwestern Polytechnical University, 710072 Xi’an, China; 3 Cogsense Technologies Limited, Berkshire RG14 1QL, UK; daniel.clarke@cogsense.co.uk

**Keywords:** cooperative localization, factor graphs, SME, SLAM

## Abstract

Precise localization is a key requirement for the success of highly assisted or autonomous vehicles. The diminishing cost of hardware has resulted in a proliferation of the number of sensors in the environment. Cooperative localization (CL) presents itself as a feasible and effective solution for localizing the ego-vehicle and its neighboring vehicles. However, one of the major challenges to fully realize the effective use of infrastructure sensors for jointly estimating the state of a vehicle in cooperative vehicle-infrastructure localization is an effective data association. In this paper, we propose a method which implements symmetric measurement equations within factor graphs in order to overcome the data association challenge with a reduced bandwidth overhead. Simulated results demonstrate the benefits of the proposed approach in comparison with our previously proposed approach of topology factors.

## 1. Introduction

The large-scale proliferation of digital technology has resulted in an increasing interest in sensors in the environment. With improvements in vehicle-to-vehicle (V2V) and vehicle-to-infrastructure (V2I) technologies, these sensors are increasingly connected through real-time communication systems. Hence, the task of cooperative localization (CL) poses a feasible solution for autonomous vehicles [1].

Cooperative localization is not a new concept. Roumeliotis et. al. and Karam et al. [2,3] demonstrated the use the Kalman filter and its family algorithms to undertake CL. Other research has provided novel solutions, including maximum a posteriori estimation (MAP) [4], particle filters [5], Markov localization [6], split covariance intersection filter [7], and the random finite set framework (RFS) [8].

As a specific example, Howard et. al. [9] use maximum likelihood estimation (MLE) to achieve the CL by combining relative measurements between robots in a least square formulation. Ahmad et. al. [10] do the same but also introduces moving landmarks to provide an improved estimate. Gulati et. al. [11] formulate the CL as a graphical model, adding a topology factor to govern the inter-node distance within a system. This is implemented as a factor graph within the Georgia Tech Smoothing and Mapping (GTSAM) [12] framework.

Successful CL depends on the correct association of sensor measurements observed by different nodes. In the case where multiple targets are present, the data association must be undertaken using semantic information uniquely identifying the target, or using computationally intensive track-to-track association.

In terms of data association, various novel solutions exist to solve this problem. Fortmann et al. [13] introduced joint probabilistic data association in the early 1980s, and it is proven to be a reliable technique. It is a widely used solution and as recently as 2015 Rezatofighi et al. [14] demonstrated its derivative to solve a challenge.

Reid [15] came up with the multi-hypothesis tracking filter where each detected target is spawned into a new track. With an increase in the received measurements, the probabilities of joint hypotheses are updated recursively using all the available information. The newly created branches are then updated and the dead branches are removed based on these probabilities. Streit et. al. [16] proposed a probabilistic multi-hypothesis tracking algorithm and Giannopoulos et. al. [17] extended it for the multi-sensor scenarios.

In addition to the concepts discussed above, further techniques exist for estimating the measurement-to-track association as part of the state estimation process. Mahler successfully addressed the issue by extending the constant-gain Kalman filter for the multi-target environments using the probability hypothesis density (PHD) filter [18]. Kamen [19] proposed an symmetric measurement equation (SME) filter for multiple target tracking based on symmetric measurement equations. Zhang et. al. [20] further use the SME Filter and topology to perform the CL.

However, within the frameworks presented by most of the techniques mentioned above, the computational complexity increases exponentially with the number of targets (and hence becomes a non-linear system). Many of the state-of-the-art approaches fuse the measurements from an external sensor to get the final state estimates for the CL, performing both data association and coordinate transformation. In our previous work [11] we successfully avoid the task of data association and coordinate transformation by adding the sum of inter-vehicular distances as constraints between the various vehicles for each state.

The concept in [11] can be better understood by an example system of three vehicles, $V_{1},V_{2}$ and $V_{3}$, in the field of view of an external sensor like RADAR. If the inter-vehicle distance between $V_{i}$ and $V_{j}$ is $d_{ij}$, then $d_{12}$, $d_{23}$ and $d_{31}$ are the possible inter-vehicle distances for the system. Important properties of the distance $d_{ij}$ are: (1) it is a scalar value, independent of the coordinate system of the censor; and (2) the sum of all the $d_{ij}$ is independent of the track-to-measurement association. Now at any given state we can form a geometric topology by connecting all the participating vehicles. Therefore, to address the challenge of data association and coordinate transformation, we convert the original measurement into the constraint of a topology factor consisting of sum of all the inter-vehicular distances. Our proposed solution performs better than the optimal Kalman filter and simultaneously scales optimally. However, formulating the topology factor results in a loss of information. This is because the topology factor converts any two participating vehicles’ coordinate positions into an inter-vehicle distance. Therefore we have lost the actual location, as the resulting scalar value has no position information.

In this paper we propose the use of the SME constraint factor, which adds full information into a factor graph to perform CL for semi or fully autonomous vehicles That is, it uses the measurements without calculating the difference, in a factor graph to perform CL for semi or fully autonomous vehicles. Our simulation indicates that the new SME factor performs better than the topology factor. In the process we address the challenge of data association and do not require an expensive data association solution.

The rest of this paper is organized as follows. Section 2 defines the problem with its constraints. Section 3 gives the overview of the SME, which is followed by explanation of factor graphs and non-linear least square optimization methods in Section 4. Section 5 presents the evaluation and Section 6 concludes the paper.

## 2. Problem Description

**Figure 1:**
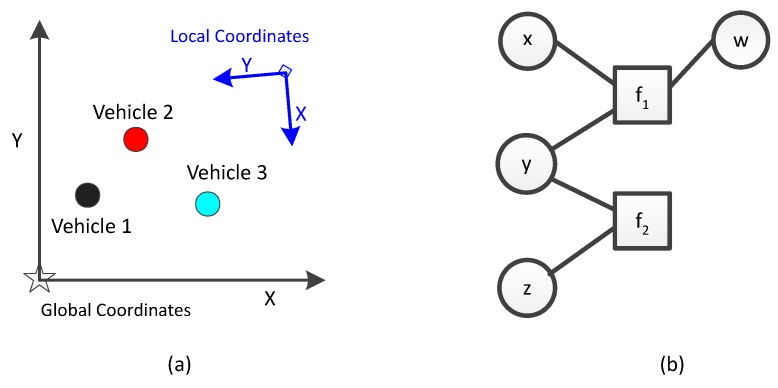
(**a**) Problem description with three vehicles. The local coordinate system is of the infrastructure sensor; (**b**) Factor graph with variables w,x,y,z and functions $f_{1}\left(w,x,y\right)$ and $f_{2}\left(y,z\right)$.

A typical scenario is shown in Figure 1a, highlighting the vehicle-infrastructure CL. The assumptions are as follows:

1. Each vehicle has an odometry and a Global Positioning System (GPS) sensor to localize itself in an absolute reference and can broadcasts its measurements.
2. The infrastructure sensor that can derive the global position of all the vehicles in its field of view, but cannot uniquely identify the vehicles. This introduces a challenge from the perspective of data association. A typical example of such sensor is a RADAR. RADAR has been extensively used by the military for surveillance [21]. Since the mid 1990s, it has been researched as an active [22] or passive [23] component of the Intelligent Vehicle Highway System (IVHS). Recently however, because of lowering of costs, it has gained a lot of traction as an infrastructure sensor for smart highways [24,25,26,27].
3. The vehicles and the infrastructure sensor can communicate in both directions without any timing delay or data error.
4. The environment has no clutter and there are no missed detections.

The the goal of vehicle-infrastructure CL is to improve the precision of position estimates of the participating vehicles. Our goal of CL is achieved by fusing the measurements from internal sensors (Assumption 1) with the measurements from an external sensor (Assumption 2). To address the data association challenge from the external sensor (Assumption 2) we use symmetric measurement equations (SMEs). The concepts of SMEs are introduced in Section 3. To represent the problem of CL we use a graph-based Simultaneous Localization and Mapping (SLAM) approach, implemented within factor graphs and use non-linear least square optimization to optimize the graph for generating the posterior state estimates. The concepts of factor graphs and non-linear least square optimization are introduced in the Section 3.

## 3. Symmetric Measurement Equations (SMEs)

As mentioned in the problem description, SMEs are primarily used to avoid the data association for the external sensor. This is possible because we use SMEs to generate ’pseudo-measurements’ using symmetric functions from the original measurements [19]. This is achieved by adding or multiplying the original sensor measurements to generate new measurements having values from all the targets. The resulting measurements are independent of the targets, thereby avoiding a computationally expensive data-association step.

The above can be understood by considering the case for two targets in one dimension. A sensor results in two measurements, $m_{1}$ and $m_{2}$. Without data association, it is not possible to associate the measurements with any of the targets. However, a pseudo-measurement $pm_{1}$ can be generated by adding the original sensor measurements as [$m_{1}+m_{2}$]. Another pseudo-measurement $pm_{2}$ can be generated by adding the values resulting from squares of the original sensor measurements as [$m_{1}^{2}+m_{2}^{2}$]. Now these two new sets of measurements [$pm_{1}$, $pm_{2}$] are called sum-of-powers. The second pseudo-measurement $pm_{2}^{\prime }$ can also be generated by multiplying the original sensor measurements as [$m_{1}\cdot m_{2}$]. These two new sets of measurements [$pm_{1}$, $pm_{2}^{\prime }$] are called the sum-of-product.

Similarly following are the two SME representations of the three targets in one dimension:

- Sum-of-powers:
  (1) $$S_{pow}=\left[\begin{array}{c} m_{1}+m_{2}+m_{3}\\ m_{1}^{2}+m_{2}^{2}+m_{3}^{2}\\ m_{1}^{3}+m_{2}^{3}+m_{3}^{3} \end{array}\right]$$
- Sum-of-product:
  (2) $$S_{prod}=\left[\begin{array}{c} m_{1}+m_{2}+m_{3}\\ m_{1}m_{2}+m_{2}m_{3}+m_{1}m_{3}\\ m_{1}m_{2}m_{3} \end{array}\right]$$

where $m_{i}$ is the measurement from the ith target.

As can be seen from the above examples, the original participating measurements result in the same number of ’pseudo-measurements’ which are independent of the original measurements. However, this also increases the non-linearity of the system as the new equations are now of the nth degree for *n* targets, square for the two- and cubic for the three-target system. Thus, we trade one challenging problem of ’data association’ with another of non-linearity. The next section describes the concept of ’factor graphs’ which we use to optimize the joint probability function and address the traded in challenge.

## 4. Non-Linear Least Square Optimization

Factor graphs are used as a framework for addressing the problem described in Section 2. They are a graphical notation and lend themselves to logically represent the problem herein. After formulating as graphs we use a non-linear least square optimization to extract the estimated state vectors. In this section we provide brief overview of these methodologies and how we use them to achieve our goal.

### 4.1. Factor Graphs

A factor graph is a bipartite graph $G_{k}=\left(F_{k},V_{k},E_{k}\right)$ with two types of nodes: factor nodes $f_{i}\in F_{k}$ and variable nodes $v_{j}\in V_{k}$. Edges $e_{ij}\in E_{k}$ can exist only between factor nodes and variable nodes, and are present if and only if the factor $f_{i}$ involves a variable $v_{j}$ [28]. They can also represent the probabilistic graphical models (PGM) and are used to implement Bayesian networks [29] and Markov random fields [30].

Figure 1b is an example of a factor graph with variables w,x,y and *z*, and functions $f_{1}$ and $f_{2}$ with factorization: $f\left(w,x,y,z\right)=f_{1}\left(w,x,y\right)*f_{2}\left(y,z\right)$. Using PGM, the example can also be represented as P(w,x,y,z)=P(w,x,y)∗P(y,z). Similarly we use the factorized probability distribution to represent the entire trajectories of all the participating vehicles as an optimization problem. The localization can then be represented by estimating the trajectory $x=\{x_{i}|i\in 0,\ldots ,n\}$, for a given set of measurements from various sensors. For example, if we have three different sensors, odometry, sensor 1, and sensor 2, then the measurements from the chosen sensors can be written as (here we choose only odometry as known sensor since we want to calculate the trajectory; others could include any form of sensors such as GPS, RADAR, LIDAR, camera):

- from the odometry sensor, $u=\{u_{i}|i\in 0,\ldots ,n\}$
- from the generic sensor 1, $z^{1}=\left\{z_{i}^{1}|i\in 0,\ldots ,n\right\}$
- from the generic sensor 2, $z^{2}=\left\{z_{i}^{2}|i\in 0,\ldots ,n\right\}$

Thus, the joint density for the data obtained from the above three sensors can be represented as:

(3) $$P\left(x,z^{1},z^{2},u\right)\propto P\left(x_{0}\right)\prod_{i}^{n}P\left(x_{i+1}|x_{i},u_{i}\right)\prod_{k}^{m}P\left(z_{k}|x_{ik}\right)$$

where $z_{k}\in \left\{z^{1},z^{2}\right\}$ denotes the measurement, originating from either sensor 1 or sensor 2.

Now the factorization is undertaken based on Gaussian distributions for the process and measurement models as:

(4) $$\begin{array}{c} x_{i}=f_{i}\left(x_{i-1},u_{i}\right)-w_{i}\Leftrightarrow P\left(x_{i+1}|x_{i},u_{i}\right)\propto \exp(-\frac{1}{2}\left|\right|f_{i}(x_{i-1}-x_{i}{\left|\right|}_{\Gamma_{i}}^{2}) \end{array}$$

(5) $$\begin{array}{c} z_{k}=h_{k}\left(x_{ik}\right)-v_{k}\Leftrightarrow P\left(z_{k}|x_{ik}\right)\propto \exp(-\frac{1}{2}\left|\right|h_{k}\left(x_{ik}\right)-z_{k}{\left|\right|}_{\Sigma_{k}}^{2}) \end{array}$$

where *h* and *f* denote the measurement and process models, and *v* and *w* are the corresponding noises with covariance matrices $\Sigma_{k}$ and $\Gamma_{i}$.

In this paper, the goal is to calculate the maximum likelihood estimate (MLE) by using the non-linear least square method:

(6) $$\begin{array}{c} \bar{\theta }=\mathrm{argmax}P\left(\theta |z,u\right)=\mathrm{argmin}\{\sum_{i=1}^{n}\left|\right|f_{i}\left(x_{i-1}-u_{i}\right)-x_{i}{\left|\right|}_{\Gamma_{i}}^{2}+\sum_{k=1}^{m}\left|\right|h_{k}\left(x_{ik}\right)-z_{k}{\left|\right|}_{\Sigma_{k}}^{2}\} \end{array}$$

The above method can be extended for any number and type of sensors. In terms of factor graphs, factors are a synonym for this measurement model. For a Gaussian noise model, the corresponding measurement factor for any Sensor−S from Equation (5) can be written as:

(7) $$\begin{array}{c} f^{Sensor-S}\left(X_{k}\right)=d\left[h_{k}\left(X_{k}\right)-z_{k}\right] \end{array}$$

where $h_{k}$ is the measurement model as the function of state variable $X_{k}$, $z_{k}$ is the measurement from the sensor and operator d(·) represents the cost function.

The first assumption for the problem description in Section 2 is the availability of odometry and GPS measurements directly from the participating vehicles. The second assumption is the availability of infrastructure sensor measurements, provided naively without any knowledge of the relatve correlation or association. As explained in Section 2, we make use of SMEs on the measurements obtained from the external sensor to solve the problem of data association. As explained above, various sensor measurements are represented as factors in a factor graph. Therefore, let us take a look at the formulations of various measurements as factors for the factor graph.

### 4.2. Odometry Factor

For a constant velocity model, the measurement equation for an odometry factor is given by:

(8) $$z_{t}^{o}=h^{o}\left(z_{t-1}^{o}\right)+n^{o}$$

where $h^{o}$ is the function to calculate the odometry measurement at time *t*, $z_{t}^{o}$ is the measurement at time *t* and $n^{o}$ is the measurement noise. The error function of the binary factor $f^{ODOM}$ between the states $X_{t},X_{t-1}$ is:

(9) $$f^{ODOM}\left(X_{t},X_{t-1}\right)≜d\left(z_{t}^{o}-h^{o}\left(z_{t-1}^{o}\right)\right)$$

The odometry measurement from the sensor is used directly, hence the noise model provided by the sensor manufacture in the form of covariance matrices is used while formulating the corresponding factors.

### 4.3. GPS Factor

The GPS measurement equation is:

(10) $$z_{t}^{g}=h^{g}\left(z_{t}\right)+n^{g},$$

where $n^{g}$ is the measurement noise, and $h^{g}$ is the measurement function, providing the relation between the measurement $z_{t}^{g}$ and the position of the vehicle $z_{t}$ at time *t*. Equation (10) gives an unary factor $f^{GPS}$ for a state $X_{t}$, which is written as:

(11) $$f^{GPS}\left(X_{t}\right)≜d\left(z_{t}^{g}-h^{g}\left(z_{t}\right)\right)$$

The GPS measurement from the sensor is used directly, hence the noise model provided by the sensor manufacture in the form of a covariance matrix is used while formulating the corresponding factors.

### 4.4. SME Factor

To perform CL, the node running the fusion receives the following:

- The odometry and GPS measurements from all/other vehicles;
- Absolute positions, in global coordinates, of all vehicles in the field of view of a configured RADAR. Here by configuration of RADAR we imply that it knows its position in global coordinates and hence is able to perform a coordinate transformation of the measurements of the detected targets in its local coordinates to the global coordinates.

As the RADAR does not perform any data association, it will not associate the calculated positions to the individual vehicles. To incorporate such information in the factor graph, we construct the SME factor. Using (1) of the SME transformation, we convert the received values from RADAR for *n* vehicles at time *t*, as:

(12) $$z_{t}^{s_{x}}=\left[\begin{array}{c} \sum_{i=1}^{n}x_{i}\\ \sum_{i=1}^{n}x_{i}^{2}\\ \cdots \\ \sum_{i=1}^{n}x_{i}^{n} \end{array}\right]+n^{sme}=h^{s_{x}}\left(x_{0},\cdots ,x_{n}\right)+n^{sme}$$

where $x_{i}$ is the absolute position received from RADAR in the *x* dimension for the ith vehicle, $h^{s_{x}}$ is the new measurement function and $n^{sme}$ is the measurement noise. Similarly *y* can be written as:

(13) $$z_{t}^{s_{y}}=h^{s_{y}}\left(y_{0},\cdots ,y_{n}\right)+n^{sme}$$

where $y_{i}$ is the absolute position received from RADAR in the *y* dimension for the ith vehicle. $h^{s_{y}}$ is the new measurement function and $n^{sme}$ is the measurement noise.

Equations (12) and (13) result in the following N-ary factor:

(14) $$\begin{array}{c} f^{SME}\left({\left(X_{0},\cdots ,X_{N}\right)}_{t}\right)≜d\left(z_{t}^{s}-h^{s}\left(z_{0},\cdots ,z_{N}\right)\right) \end{array}$$

**Figure 2:**
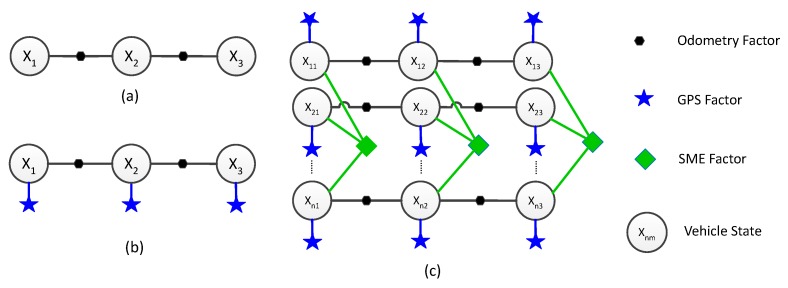
(**a**) Factor graph for a single vehicle with 3 state nodes and 2 odometry factors; (**b**) Factor graph for a single vehicle with 3 state nodes, 2 odometry factors and 3 GPS factors; (**c**) Factor graph for n vehicles with 3 state nodes each, 6 odometry factors, 9 GPS factors and 3 symmetric measurement equation (SME) factors.

Figure 2 shows factor graphs with different kinds of factors.

The SME measurement can be considered a pseudo measurement, hence we must also calculate a covariance matrix for it.

If $\sigma_{x}^{2}$ and $\sigma_{y}^{2}$ are the *x* and *y* variances, respectively, for the RADAR, then we have:

(15) $$Cov\left(x,y\right)=\mathrm{diag}\left[\begin{array}{c} \sigma_{x_{1}}^{2},\cdots ,\sigma_{x_{n}}^{2},\sigma_{y_{1}}^{2},\cdots ,\sigma_{y_{n}}^{2} \end{array}\right]$$

We follow [31] where the covariance for the new SME measurement can be defined as:

(16) $$\sigma_{sme_{x,y}}^{2}=M\cdot Cov\left(x,y\right)\cdot M^{T}$$

where *M* is a 1X2N matrix as follows:

(17) $$M=\left[\begin{array}{c} \frac{\partial }{\partial x_{1}}\left(z_{t}^{s}\right),\cdots ,\frac{\partial }{\partial x_{n}}\left(z_{t}^{s}\right),\frac{\partial }{\partial y_{1}}\left(z_{t}^{s}\right),\cdots ,\frac{\partial }{\partial y_{n}}\left(z_{t}^{s}\right) \end{array}\right]$$

### 4.5. Smoothing

The non-linear problem formulated using the factor graph is solved using the Levenberg Marquardt linearization algorithm. This is a Gauss–Newton style non-linear optimizer. Using an initial estimate $x_{0}$ it iteratively finds an update Δ from the linearized system:

(18) $$\underset{\Delta }{arg min}J\left(x_{0}\right)\Delta -b\left(x_{0}\right)$$

where $J(x_{0})$ is the sparse Jacobian matrix at the current linearization point $x_{0}$ and $b\left(x_{0}\right)=f\left(x_{0}\right)-z$ is the residual for given the measurement *z*. The Jacobian matrix is equivalent to a linearized version of the factor graph, and its block structure reflects the structure of the factor graph. After solving (18), the linearization point is updated to the new estimate $x_{0}+\Delta$. Further details on this process are presented within [32].

As seen in Equation (18) we need to calculate the Jacobians to obtain the optimal state. The Jacobian for the odometry is calculated from the Equation (8). Now, the measurement $z_{t}^{o}$ in the equation represents *x* and *y* position in a 2−D plane. As such, Equation (8) becomes,

(19) $${\left(x,y\right)}_{t}^{o}=h^{o}\left({\left(x,y\right)}_{t-1}^{o}\right)+n^{o}$$

The Jacobian for $h^{o}\left(x,y\right)$ (constant velocity is a linear function) is obtained with ∂x and ∂y as:

(20) $$J^{O}=\frac{\partial (h^{o}\left(z^{o}\right)+n^{o})}{\partial x\partial y}=\frac{\partial (h^{o}\left(x,y\right))}{\partial x\partial y}=\left[\begin{array}{cc} \frac{\partial (h^{o}\left(x,y\right))}{\partial x} & 0\\ 0 & \frac{\partial (h^{o}\left(x,y\right))}{\partial y} \end{array}\right]=\left[\begin{array}{cc} 1 & 0\\ 0 & 1 \end{array}\right]$$

Similarly the Jacobian used for the GPS factor can be calculated from Equation (10) and is same as that of the odometry.

From Equations (12) and (14), it can be seen that for the *N* participating vehicles, there will be a total of *N* factors between the state nodes. The Jacobian for each of them ($J_{1}^{S},\cdots ,J_{N}^{S}$) can be written as:

(21) $$J_{1}^{S}=\left[\begin{array}{cc} \frac{\partial (\sum_{i=1}^{N}\left(x_{i}\right))}{\partial x} & 0\\ 0 & \frac{\partial (\sum_{i=1}^{N}\left(y_{i}\right))}{\partial y} \end{array}\right],J_{2}^{S}=\left[\begin{array}{cc} \frac{\partial (\sum_{i=1}^{N}\left(x_{i}^{2}\right))}{\partial x} & 0\\ 0 & \frac{\partial (\sum_{i=1}^{N}\left(y_{i}^{2}\right))}{\partial y} \end{array}\right],\cdots ,J_{N}^{S}=\left[\begin{array}{cc} \frac{\partial (\sum_{i=1}^{N}\left(x_{i}^{N}\right))}{\partial x} & 0\\ 0 & \frac{\partial (\sum_{i=1}^{N}\left(y_{i}^{N}\right))}{\partial y} \end{array}\right]$$

This can be generalized as:

(22) $$J_{p}^{S}=\left[\begin{array}{cc} \frac{\partial (\sum_{i=1}^{N}\left(x_{i}^{p}\right))}{\partial x} & 0\\ 0 & \frac{\partial (\sum_{i=1}^{N}\left(y_{i}^{p}\right))}{\partial y} \end{array}\right]\forall p=1\cdots N$$

## 5. Evaluation

### 5.1. Simulation Setup

**Figure 3:**
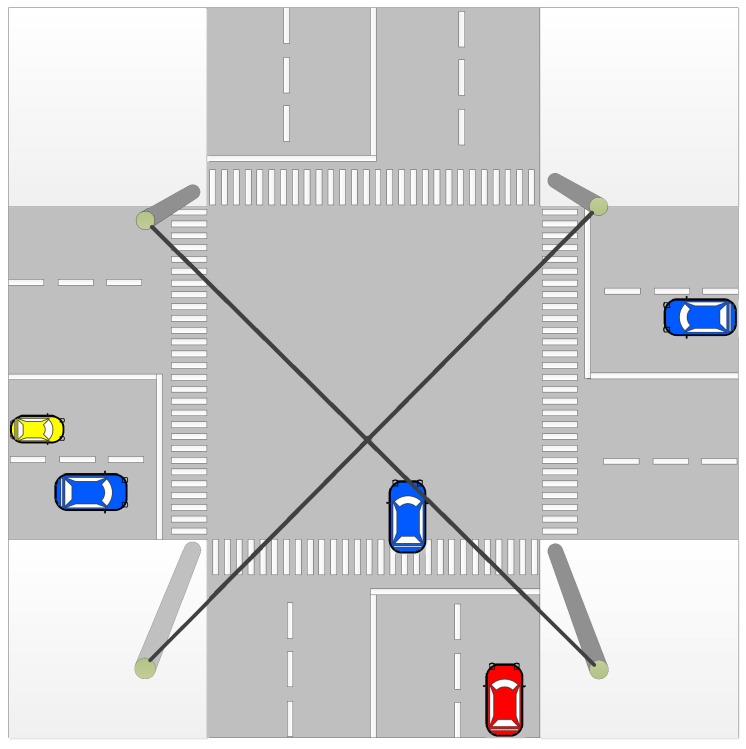
Simulation scenario of an intersection with five vehicles. The simulated RADAR is mounted above the ground (represented with four poles) on the cross section. The RADAR is assumed to have $360^{\circ }$ field of view. The vehicles represent the state of their trajectory at random time *t*.

To address the primary challenge of the data association or the track-to-measurement association, the data from internal sensors from multiple vehicles and external infrastructure sensor is naively shared between various participating nodes and the fusion node. Unlike the other methods the proposed methodology does not require the corresponding covariances to be exchanged. Based on these requirements, we define the following scenario sets for an evaluation:

1. Two vehicles with random trajectories on a ground plane with an infrastructure RADAR.
2. Three vehicles with a constant turn model on a ground plane with an infrastructure RADAR.
3. An intersection with five vehicles with an infrastructure RADAR mounted at the center of intersection (Figure 3). We assume the infrastructure RADAR has an equal field of view for all the four directions. This test uses the trajectories with a constant velocity model.
4. Monte Carlo simulations for 1000 iterations for 2, 3 and 4 vehicles. This test also reflects the trajectories with a constant velocity model.

We simulate each of the sets for the 200 steps. The above sets are further divided into one with and one without GPS sSensor measurements. Georgia Tech Smoothing and Mapping (GTSAM) [12] is used to implement the factor graphs.

Simulated vehicles provide their own odometry measurements and the location in global coordinates. Infrastructure RADAR provides global coordinates for the vehicles in its field of view without performing any data association. The covariances are assumed as diag[1.0,1.0], diag[10.0,10.0] and diag[0.5,0.5] for odometry, GPS and RADAR, respectively.

Results from the simulation of the two sets are compared three ways, between:

1. the fused trajectory only using odometry;
2. the fused trajectory for odometry and topology factor [11]; and
3. the fused trajectory for the odometry and SME factor (proposed in this work).

As stated before, the experiments are performed with and without a GPS factor (constructed from GPS measurements), therefore a similar three-way comparison is performed with GPS measurements too.

The performance is measured by calculating root-mean-square error (RMSE) for the complete system. The total error is the sum of the RMSE of each vehicle for *n* steps:

$$Error=\sqrt{\frac{\sum_{j=1}^{n}\sum_{i=1}^{2}{\left[{\left(x_{i_{est}}-x_{i_{real}}\right)}^{2}+{\left(y_{i_{est}}-y_{i_{real}}\right)}^{2}\right]}^{j}}{n}}$$

### 5.2. Results

**Figure 4:**
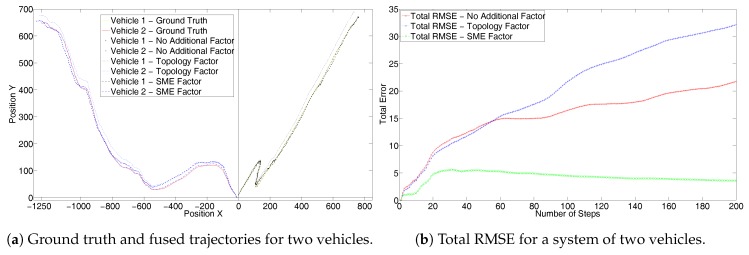
First set without GPS for two randomly simulated trajectories.

Figure 4a shows the results from the first set for two vehicles, each with a random trajectory and without GPS measurements. Figure 4b shows the corresponding total system RMSE. It can be seen that the trajectories generated by using the SME filter are closer to the ground truth. Also, it can be seen that the total system RMSE with SME factors is far superior than the other two. In fact, only odometry performs better than the topology factor. This is because the topology factor adds only relative inter-vehicle constraints and does not consider the true position. As the RADAR has a lower error covariance, the topology measurements resulting from it have a higher weight and forces the graph to converge to an incorrect solution. Hence, this results in a negative performance. On the other hand, the SME factor incorporates full information as it uses the full global coordinates obtained from the RADAR and hence gives better performance.

**Figure 5:**
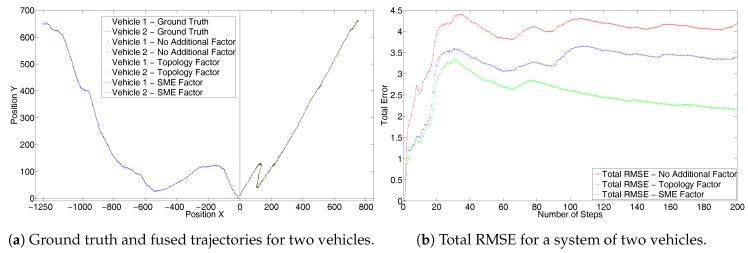
First set with GPS for two randomly simulated trajectories.

Figure 5a also includes the GPS measurement for the same set of trajectories as of Figure 4a. Figure 5b shows the corresponding RMSE. Here after fusing of the GPS sensor information, the topology factor performs better than the one with only odometry and GPS. This happens because GPS information forces the topology to converge to the true position. However, the GPS information further improves the results from the SME factor and its performance remains superior than for the other two.

**Figure 6:**
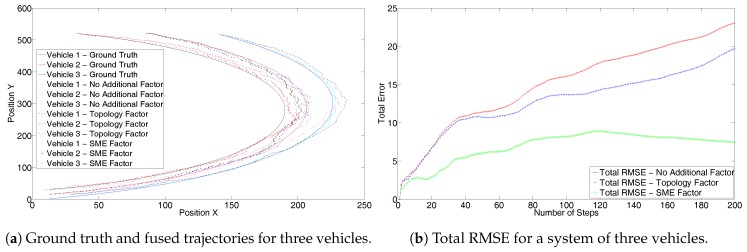
Second set without GPS for three trajectories with constant turn model.

**Figure 7:**
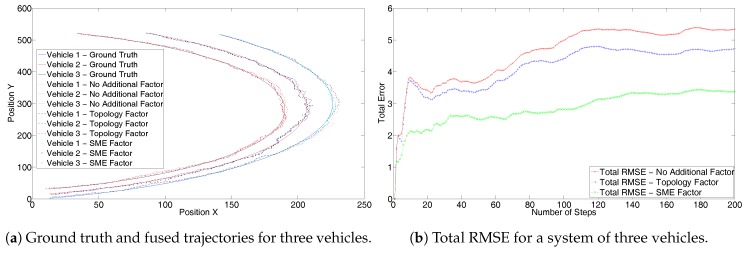
Second set with GPS for three trajectories with constant turn model.

Next, the effectiveness of the solution can be seen from the second scenario of three vehicles using a constant turn model. Figure 6a shows the results without the GPS sensor. These are similar to the Figure 4a. The trajectory generated using the SME filter is closer to the ground truth. Due to the nearness of the trajectories of the three vehicles in the system, the result can be better analyzed from the corresponding RMSE values, as highlighted in Figure 6b. The RMSE also shows similar performance to that in Figure 4b. Figure 7a shows results with the GPS sensor information and Figure 7b shows the corresponding complete system RMSE. Both the trajectories and the RMSE in Figure 7 show similar characteristics to those of Figure 5.

**Figure 8:**
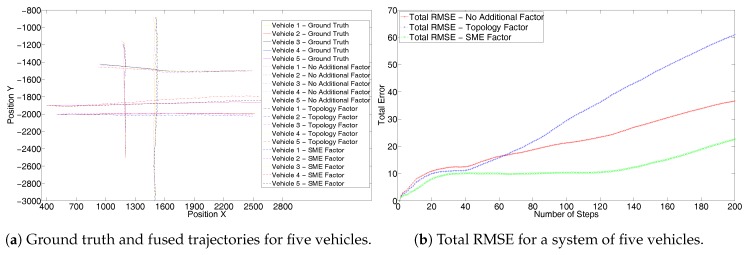
Third set without GPS for the simulated trajectories of five vehicles at an intersection.

**Figure 9:**
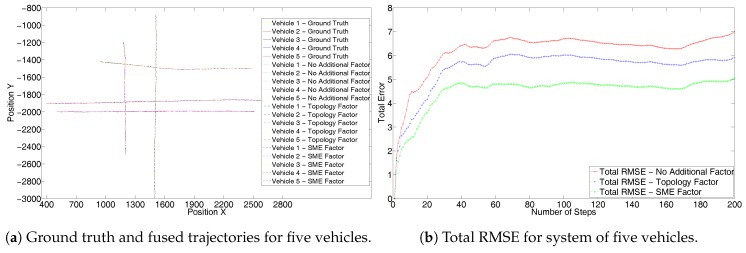
Third set with GPS for the simulated trajectories of five vehicles at an intersection.

To further evaluate and demonstrate the scalability of the proposed solution we simulate the third scenario of an intersection as modeled in Figure 3. Here the vehicles travel with a constant velocity. Figure 8a shows the results without the GPS sensor. Again it can be seen that as in Figure 4a, trajectories resulting from the use of an SME factor are closer to the ground truth. Due to a large number of trajectories in the system, the result can be better analyzed from the corresponding RMSE values in Figure 8b. The RMSE also shows superior performance of the SME factor to that of Figure 4b. Figure 9a shows results with the GPS sensor included and Figure 9b shows the corresponding complete system RMSE. Again these show that the SME factor outperforms the other two as seen in Figure 5 and Figure 7.

Lastly we evaluate the stability of the proposed solution by running a Monte Carlo simulation of 1000 iterations for a system with 2 , 3 and 4 vehicle trajectories. We analyze the results by calculating the average RMSE of 1000 iterations for each of the system as shown in Table 1.

**Figure 10:**
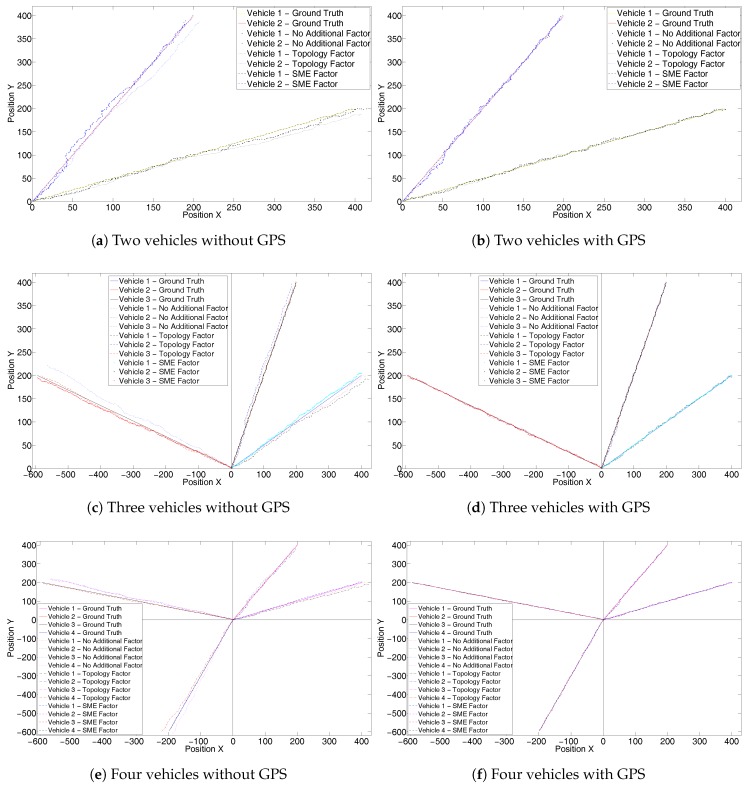
One iteration of ground truth and fused trajectories for 2-, 3- and 4-vehicle systems for Monte Carlo simulation of 1000 iterations

Figure 10a–f shows one set of trajectories of the 1000 iterations performed as part of Monte Carlo simulation. It can be observed that the trajectories with an SME factor for 2-, 3- and 4-vehicle systems are closer to the ground truth than the other two. Table 1 shows clear reduction of RMSE error in localization with an addition of the SME factor. However, it also highlights an anomaly for the two-vehicle system, when the SME factor without GPS performs a little better than the one with GPS. However, the difference is very small and rest of the results show considerable improvements with the GPS factor.

### 5.3. Plug and Play and Online Execution

All the previous tests show the validity and stability of the proposed solution of SME factors. However, it has been demonstrated using an offline batch Levenberg Marquardt optimizer. We optimized the full graph and assumed that the measurements from all the sensors were available all the time. However, in a real scenario, all the measurements may not be available all the time (for example from the external sensors). They may be only available at run time and for only a small duration (when the vehicles are in the field of view of the external sensor). In that case, the system should be able to automatically incorporate such measurements to calculate the final state estimates. Such scenarios can also be classified as ’plug and play’. The ’plug’ phase adds the new, previously unseen, measurements at the run time when they are available. The ’play’ phase fuses the newly added measurements and results in a better state estimate of the participating vehicles. When the measurements are no longer available the system automatically uses the remaining available measurements.

In such real scenario an online incremental smoothing algorithm like iSAM2 [33] (supported in GTSAM) is required. Further, the GTSAM framework supports the above described plug and play feature [34] thereby providing a efficient platform for such scenarios.

As we have seen, the SME factor is also quite efficient when GPS is unavailable. Hence, it can be used in scenarios like underground parking lots and tunnels when there is no line of sight to the satellites. Also, in scenarios like urban canyons where a reduced number of satellites are available in direct line of sight, addition of the SME factor would improve the CL. This is because the SME factor is formulated from the measurements of a precise infrastructure sensor and hence has a lower error covariance. Therefore, the SME factor has a higher weight during smoothing and arrives at a more precise solution.

In this section we demonstrate a simple scenario of two vehicles on a ground plane for 200 steps. For the first 50 steps the vehicle is assumed to have GPS measurements. At the 51st step it enters a tunnel and loses the GPS connection. At the 151st step it exits the tunnel and GPS is restored without any delay. The tunnel has an infrastructure RADAR which is configured to provide the global coordinates of the vehicles in its field of view. We assume RADAR does not perform any data association.

**Figure 11:**
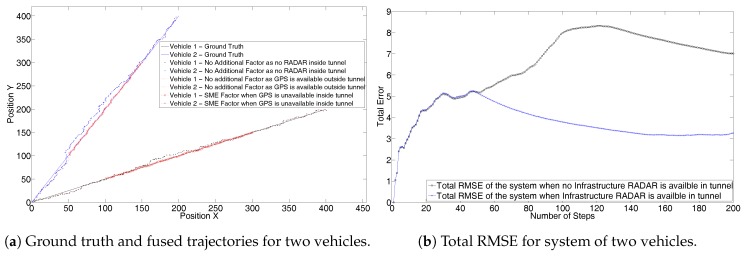
Simulation of two vehicles through the tunnel, demonstrating the use of SME factor using online iSAM2 algorithm.

Figure 11a shows the trajectories of the two vehicles. For the case when the tunnel does not have a RADAR sensor, and hence no SME factor, we can see the fused trajectories of the vehicles drift farther from the ground truth after they enter the tunnel. However, when the tunnel is equipped with the infrastructure RADAR and the SME factor is added as the constraint between the trajectories when the vehicle is inside the tunnel, the fused trajectories remain closer to the ground truth. The trajectory resulting from the SME factor is shown in the red color. For the scenarios with and without the RADAR, the first and last 50 steps have odometry and GPS as factors. Inside the tunnel, without the RADAR, only odometry factors are added to the graph. However, with RADAR, odometry and SME factors are added in the graph. This additional information in form of the SME factor helps in convergence to the true position.

The result can also be analyzed from the RMSE of the total system shown in Figure 11b. Through to the 50th time step, both the systems have the same RMSE values. Starting at 51st time step the RMSE error increases for the system which does not have the infrastructure RADAR. However, for the system with the infrastructure RADAR the RMSE falls as RADAR has a lower error covariance.

### 5.4. Final Remarks

In our simulation experiments, original measurements from the RADAR have been used, hence data association is avoided. Also, the original measurements are used to construct the new SME measurements, therefore no information is lost during transformation. Furthermore, the use of factor graph helps in addressing the non-linearity of the systems efficiently. Hence, it scales well with the degree of equations for SME factors, which directly depend on the number of vehicles in the field of view of the RADAR. To perform CL, traditional distributed Gaussian-based fusion methods using Kalman filter, extended Kalman filter, PHD filter [2,3,18], etc. require states and covariances to be sent on the network with each time step to the node performing the fusion. However, for the proposed method only measurements are sent, keeping the bandwidth requirements to minimum.

We successfully avoided the measurement to track association issues in RADAR measurements, albeit with the assumption of a clutter-free environment. In practice this is not the case. Incorrect values in the graph degrade the optimization [35]. Therefore, clutter for the factor graphs can be handled at two levels, either actively at the RADAR [36,37,38], or later during the optimization of factor graphs [39,40]. These ideas can be further explored to tackle the challenges of the clutter.

Although the SME factor performs better than the topology factor, using topology factor we are also able to avoid coordinate transformations. Since we need the absolute position to construct the SME factor, we also require coordinate transformations. Static infrastructure sensors are generally configured only once and hence the coordinate transformation is only a fixed time process.

## 6. Conclusions

In this paper we present a novel approach to the challenge of vehicle-infrastructure cooperative localization using a combination of factor graphs and symmetric measurement equations. Specifically, these techniques address the challenges of track-to-measurement association, bandwidth expense and computational scalability which are usually associated with this task. The work presented herein provides improved accuracy over our previous work which added a unique topology factor to the factor graph. However, it should be noted that this work is unable to resolve the coordinate transformation issue which is addressed using the topology factor.

The simulations show that even in the absence of GPS measurements (like in tunnels, urban canyons and underground parking lots), the SME factor remains robust and performs better than the topology factor. The plug and play framework allows the sensor measurements to be incorporated online from sensors outside the ego vehicle. With the use of the factor graphs, the rising non-linearity of the system with an increase in the number of the agents is adequately addressed and the use of the incremental smoothing algorithms ensures that the system remains computationally efficient.

The solution proposed herein has the potential to address some of the major challenges for highly assisted and autonomous vehicles. Specifically, this work holds significant potential in applications where vehicles cooperate with external agents, such as sensors on other vehicles and sensors mounted within the infrastructure in order to improve their localization accuracy. Further research should consider how different sensors such as inertial and wheel encoders can be incorporated into the cooperative localization model, and how external targets can be localized and tracked within this framework. Finally, this work has not yet considered some real-world issues such as clutter, target birth/death and missed detections, which should be investigated in any future work which seeks to test these techniques on real world data.

## Figures and Tables

**Table 1:** Average RMSE for 1000 iterations.

Number	No Additional Factor		Topology Factor		SME Factor	
of Vehicles	Without GPS	With GPS	Without GPS	With GPS	Without GPS	With GPS
2	19.148325	4.443266	14.525345	3.167303	1.266813	1.291505
3	23.784680	5.462410	26.036128	4.530059	6.811776	2.510663
4	27.664985	6.314271	30.067669	5.490308	6.145480	2.694074
